# Can video streaming improve first aid for injured patients? A prospective observational study from Norway

**DOI:** 10.1186/s12873-024-01010-0

**Published:** 2024-05-28

**Authors:** Siri Idland, Jo Kramer-Johansen, Håkon Kvåle Bakke, Milada Hagen, Kristin Tønsager, Hans-Christian Stoud Platou, Magnus Hjortdahl

**Affiliations:** 1 Faculty of Health Sciences, Department of Nursing and Health Promotion, Faculty of Health Science, Oslo Metropolitan University, Oslo, Norway; 2 Division of Prehospital Services, Oslo University Hospital, Oslo, Norway; 3 Institute of Clinical Medicine, University of Oslo, Oslo, Norway; 4 Department of Anaesthesia and Critical Care, University Hospital of North Norway, Tromsø, Norway; 5 Department of Health and Care Sciences, Faculty of Health Science, UiT, The Arctic University of Norway, Tromsø, Norway; 6 Air Ambulance Department, Stavanger University Hospital, Pre-hospital Division, Stavanger, Norway; 7 The Norwegian Air Ambulance Foundation, Oslo, Norway; 8 Department of Health Studies, University of Stavanger, Stavanger, Norway; 9 Division of prehospital services, Vestre Viken Health Trust, Drammen, Norway

**Keywords:** First aid, Injury, Video streaming, EMCC

## Abstract

**Background:**

Video streaming in emergency medical communication centers (EMCC) from caller to medical dispatcher has recently been introduced in some countries. Death by trauma is a leading cause of death and injuries are a frequent reason to contact EMCC. We aimed to investigate if video streaming is associated with recognition of a need for first aid during calls regarding injured patients and improve quality of bystander first aid.

**Methods:**

A prospective observational study including patients from three health regions in Norway, from November 2021 to February 2023 (registered in clinical trials 10/25/2021, NCT05121649). Cases where video streaming had been used as a supplement during the medical emergency call were compared to cases where video streaming was not used during the call. Patients were included by ambulance personnel on the scene of accident if they met the following criteria: 1. Ambulance personnel arrived at a patient who had an injury, 2. One or more bystanders had been present before their arrival, 3. One or more of the following first aid measures had been performed by bystander or should have been performed: airway management, control of external bleeding, recovery position, and hypothermia prevention. Ambulance personnel assessed quality of first aid performed by bystander, and information concerning use of video streaming and patient need for first aid measures recognized by dispatcher was collected through EMCC audio logs and patient charts. We present descriptive data and results from a logistic regression analysis.

**Results:**

Data was collected on 113 cases, and dispatchers used video streaming in addition to standard telephone communication in 12/113 (10%) of the cases. The odds for the dispatcher to recognize a need for first aid during a medical emergency call were more than five times higher when video streaming was used compared to no use of video streaming (OR 5.30, 95% CI 1.11-25.44). Overall quality of bystander first aid was rated as “high”. The odds ratio for the patient receiving first aid of higher quality were 1.82 (*p*-value 0.46) when video streaming was used by dispatcher during the call.

**Conclusion:**

Our findings show that video streaming is not frequently used by dispatchers in calls regarding patients with injuries, but that video streaming is associated with improved recognition of patients’ first aid needs. We found no statistically significant difference in first aid quality comparing the calls where video streaming as a supplement were used with the calls with audio only.

**Supplementary Information:**

The online version contains supplementary material available at 10.1186/s12873-024-01010-0.

## Background

A medical dispatcher at an emergency medical communication center (EMCC) is often the first link in the trauma chain of survival. In addition to prioritizing and dispatching resources, the dispatchers have an important role to instruct the caller in first aid [1, 2]. Identifying which first aid measures the patient needs can be difficult with audio as the only source of information, and a previous study has shown that dispatchers have a low sensitivity for correctly identifying injured patients in need of first aid [3]. Death by injury is one of the leading causes of death, and bystanders are recommended to begin lifesaving first aid measures until the arrival of professional health services [4]. It is physiologically plausible that bystander first aid for injured patients may reduce mortality, which is further backed by a small survival benefit in the few clinical studies conducted on the topic [5, 6]. Nonetheless, the scientific evidence of the relationship between bystander first aid on injured patients and patient outcome is still sparse [7]. Video streaming was introduced in the EMCCs in Norway in 2020 as an additional communication tool between caller and dispatcher [8]. The solution is live only, and no images are stored. Scientific knowledge on the effects of use of video streaming in patient treatment is sparse, but video streaming has been shown to improve bystander’s hand positioning and compression rate during cardiopulmonary resuscitation (CPR) [9]. No research has been conducted on the effects of video streaming on a trauma population.

We wanted to investigate if video streaming affected whether the dispatchers recognized the need for first aid measures in injured patients, and whether the use of video streaming could affect the quality of bystander first aid. We conducted a prospective, observational study based on the introduction of video streaming capabilities in Norwegian EMCCs.

## Methods

### Design and setting

**Fig. 1:**
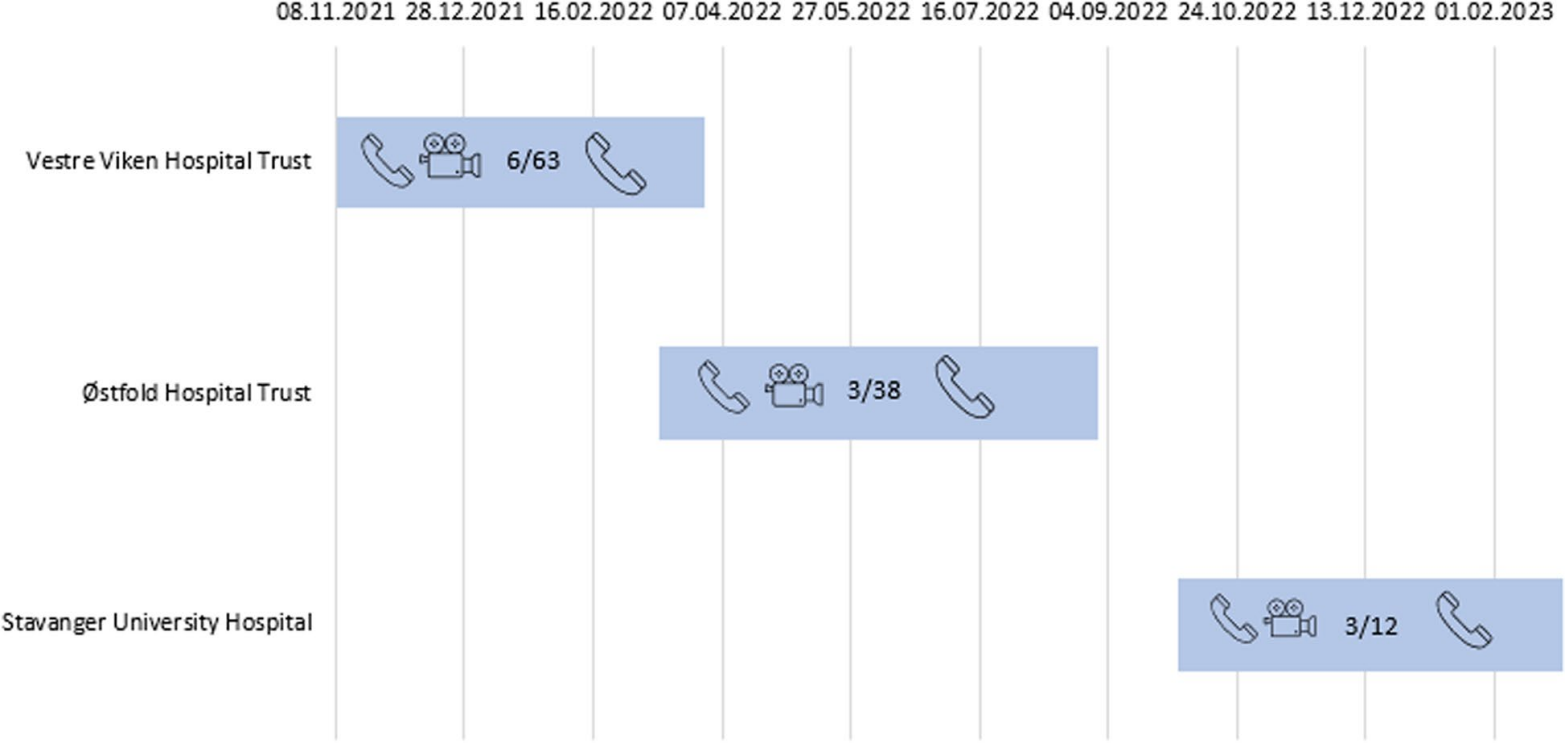
Overview of the three participating regions, timeline for inclusion of patients, number of calls with video streaming and number of patients included

The study is a prospective, observational study, assessing patients with injuries in need of bystander first aid, where bystanders had contacted the medical emergency number. Patient cases where video streaming was used as a supplement to audio during the medical emergency call were compared to cases where video streaming was not used during the call. Three health regions located in the Eastern and Western parts of Southern Norway were included in the study. The number of inhabitants of these regions is approximately 950.000. A fourth region was supposed to participate in the study but was excluded as video streaming was not implemented in the region during the study periods originally planned. The three regions included patients in the study during overlapping time periods, due to different timepoint of implementation of the video streaming solution. For overview of inclusion periods, see Figure 1. Patients were included from November 2021 to February 2023. Implementation of the solution for video streaming had been done independently from this study by each EMCC. The dispatchers at the EMCCs, who are either nurses or emergency medical technicians/paramedics, do not have algorithms for when to apply video streaming but do so at their own discretion after local procedures.

### Inclusion and data collection

Patients were included by ambulance personnel on scene, each patient generating a case. The three inclusion criteria were: 1. Ambulance personnel arrived at a patient who had an injury, 2. One or more bystanders had been present with the patient before their arrival, 3. One or more of the following first aid measures had been performed by bystanders or should have been performed: airway management, external bleeding control, recovery position and hypothermia prevention. Ambulance personnel included patients by completing an evaluation form, the First Aid Quality Assessment (FAQA) tool [10]. With the tool, they assessed each of the four first aid measures that had been performed or should have been performed and rated the quality on performed first aid measures on a Likert-scale from 1 to 5 (1- very poor quality to 5 – very high quality). Information on whether the bystander was trained or a lay person was also collected, in addition to age and gender. No standardized criteria checklist for assessing first aid quality exists, however, the FAQA-tool has been validated in a previous study [10]. Patients were excluded if they were alone and had called the EMCC themselves, or if the person who called was not with the patient. The FAQA tool was accessed by a QR-code available in the ambulance or by an icon added on the duty tablets. The tool took less than two minutes to complete on average and data was electronically transferred to a secure server (Services of sensitive data (TSD) by the University of Oslo) accessible only to study personnel.

All participating ambulance personnel were informed about the study during in-house training days and had to complete an online course prior to inclusion of patients. Total number of ambulance personnel in all three regions were approximately 1000. The study had one local contact person for each region, who regularly posted updates and information about the study in respective communication channels.

Information about the medical emergency calls, use of video streaming during the call and whether the dispatcher recognized a need for first aid measures was collected retrospectively from audio logs and EMCC patient charts. The audio logs are logs of the conversation between the caller (bystander) and dispatcher before the arrival of EMS, hence all data retrieved from audio logs contained information on the situation before the arrival of EMS. These data were collected by research assistants in the different health regions, who participated in all parts of the execution of the study in their region. Two separate forms were developed for data collection from both audio logs and EMCC patient charts (see additional file 4 and 5). All research assistants were trained by SI in completion of the form prior to data collection. Patients meeting the exclusion criteria were excluded during this retrospective data collection.

### Outcomes

The primary outcome was whether the dispatcher recognized a need for bystander first aid or not during the medical emergency call. Relevant first aid measures for this study were limited to: airway management, external bleeding control, recovery position and hypothermia prevention. For first aid measures to be registered as recognized, the audio log had to contain verbal information on bystander first aid. The secondary outcome was whether bystander first aid measures were performed with high or low quality. Performed first aid measures by bystander and quality of the measures performed were rated by ambulance personnel arriving at the scene of accident.

### Background variables

Background variables collected were age, gender, and bystander background. The variable “age” was recoded into five age groups. The variable “bystander background” collected information on whether the bystander was police/fire brigade, civilian first aid responder or a lay person. This variable was recoded into a binary variable showing whether the bystander was trained or a lay person. The variable “video streaming” consisted of three categories; “yes”, “no” and “did not function” (due to technical issues). The variable was recoded into a binary variable, where “no” and “did not function” were grouped as “no”. The study’s outcome variables used for assessment of associations, “bystander first aid quality” and “recognition of need for first aid”, were also recoded into binary variables. For bystander first aid quality, very low, low, and medium quality were recoded into the group “low quality”. High quality and very high quality were recoded as “high quality”. Recognition of need for first aid consisted of five categories: (1) Need is recognized, and bystander has started first aid measures, (2) Need is recognized, and dispatcher asks bystander to start first aid, (3) Bystander informs dispatcher that first aid is being performed before dispatcher has asked, (4) Need is not recognized, first aid measures are not performed (no measures are mentioned during the call), and (5) Uncertain. This variable was recoded into a binary variable to enable statistical analysis. Category 1, 2 and 3 was recoded as “need for first aid recognized”. Category 4 and 5 as “need for first aid not recognized”, as only three cases were registered as uncertain.

### Sample size considerations

We estimated a sample size for the study of 170 patients where video streaming had been used in addition to audio by dispatcher during the medical emergency call, and the same number for patients where audio only had been used by the dispatcher during the call. This sample size was estimated based on a hypothesis on an increase of number of cases where the dispatchers recognized a need for first aid measures from 35% (in the previous study by Bakke et. al) to 50% [3].

### Statistical analysis

Descriptive data were presented as median and range for continuous variables and counts with percentages for categorical variables. To assess possible associations between selected variables, we fitted univariate and multivariate logistic regression models. The results are presented as odds ratios (OR) with 95 % confidence intervals (CI). *P*-values <0.05 were considered as statistically significant. All statistical analysis were computed using SPSS version 28.

### Ethics

The study was registered in clinicaltrials.gov in October 2021 (NCT05121649).

We applied to the Regional Committees for Medical and Health Research Ethics (REK), the South-East B committee (reference number 247264). REK assessed and appraised the study as outside the scope of the Health Research Act, and further needed to be assessed by Sikt - the Norwegian Agency for Shared Services in Education and Research, as well as local data protection officers at each study site. Sikt assessed the study and granted approval (reference number 804294). We applied and were granted approval from local data protection officers from all study sites. Informed consent from the included patients was waived by Sikt due to the difficulty obtaining written consent from patients where time to hospital might be urgent, as well as a possibility of unconsciousness among patients at the time of arrival of the ambulance. Passive consent was still maintained, and ambulance personnel gave included patients written information about the study and how to withdraw if they did not wish to participate. A data protection impact assessment (DPIA) was developed according to procedures for passive consent. Followingly, due to the waived consent, we applied for and was given dispensation from the duty of confidentiality by the Norwegian Directorate of Health to named members of the project who collected data from audio logs and EMCC journals (21/7455).

## Results

**Fig. 2:**
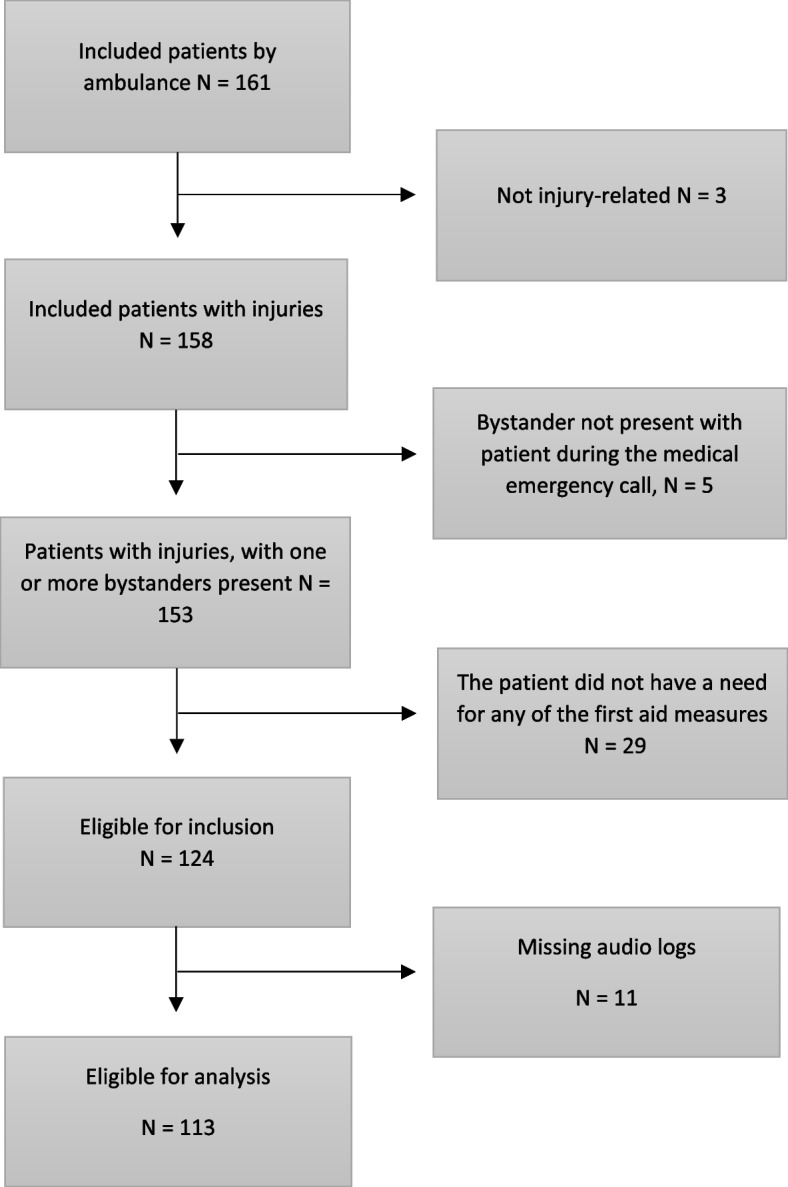
Flowchart of included patients

A total of 113 patients were eligible for analysis. Originally, 161 patients were included. Of these, five patients were excluded because either the patient himself/herself had called the medical emergency number, or the caller was not with the patient at the time of the call. Three patients were excluded due to non-injury related situations, and 29 patients were excluded because no need for first aid measures was registered (see flow chart, Fig. 2). Eleven audio logs were non-retrievable. The patient sample consisted primarily of males (58%, 65/113) and median age was 53. The lowest age was 3 years, and the oldest 98. In more than half of the cases, the bystander was a lay person. Description of the whole sample is provided in Table 1. Video streaming was used by the dispatchers in 10% (12/113) of the included cases, and details of these cases are provided in Additional file 1.

**Table 1:** Age of patient, sex of patient and bystander background (*N*=113)

	**N (%)**	**Median**	**Min, max**
**Sex of patient**			
Male	65 (58)		
**Age of patient**		56	3, 98
0-18	19 (17)		
19-30	17 (15)		
31-50	15 (13)		
51-70	35 (31)		
71<	27 (24)		
**Bystander background**			
Police/fire brigade	6 (5)		
Civilian first aid responder	40 (35)		
Lay person	67 (69)		

### Recognition of need for first aid

In the cases where audio only was used during calls, a need for first aid was recognized in 49/101 (49%) of the calls, and in the cases where video streaming was used during the call, 10/12 (83%). In total, the dispatchers recognized a need for one or more first aid measures in half (59/113, 52%) of the cases’ calls. In the remaining cases, the dispatchers did not recognize a need for first aid during the call according to the data retrieved from the audio logs. The dispatcher was more likely to recognize a need for first aid during the call when video streaming was used. When the dispatcher used video streaming, the odds for the dispatcher to recognize a need for first aid compared to not using video streaming was 5.30 (95% CI 1.11-25.44, *p*-value <0.05). The dispatchers recognized all categories of lifesaving first aid measures, and most commonly "bleeding control" and "hypothermia prevention".

In calls where video streaming was used, the dispatcher instructed on further adjustments of first aid measures in 1 of 3 performed measures (5/13 of first aid measures). When the dispatcher did not use video streaming, instruction in further adjustments of first aid measures were given by the dispatcher in 1 of 6 instructed first aid measures (10/58 of first aid measures). Descriptive statistics retrieved from audio logs are shown in Table 2.

**Table 2:** Data from review of audio logs for all cases (*N*=113). Percentage (%) calculated from the total n in each column

	**Calls with audio only (*n*=101)**	**Calls with video streaming (*n*=12)**	**All calls (*N*=113)**
	n, (%)	n, (%)	n, (%)
Need for first aid recognized by dispatcher during the call	49 (49)	10 (83)	59 (52)
Airway management performed according to the conversation	4 (4)	2 (17)	6
Bleeding control performed according to the conversation	20 (20)	4 (33)	24
Recovery position performed according to the conversation	13 (13)	4 (33)	17
Hypothermia prevention performed according to the conversation	21 (21)	3 (25)	24

### First aid measures

**Fig. 3:**
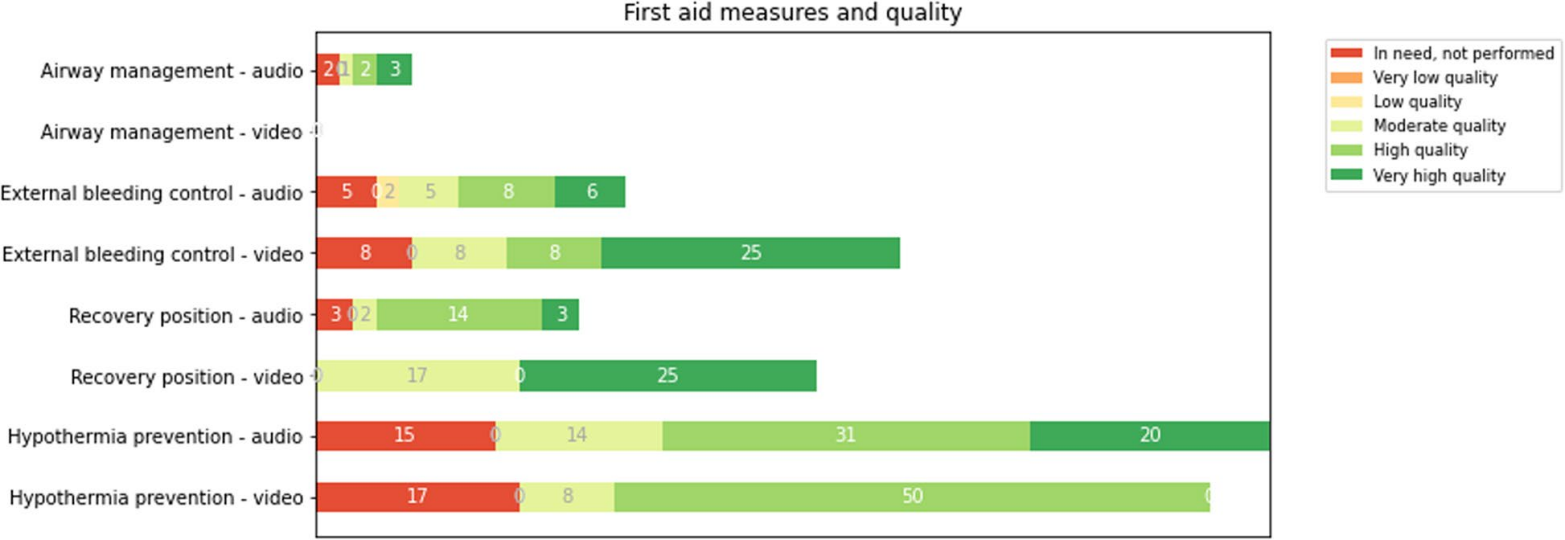
Overview in % of cases with video streaming and with audio only: First aid measures performed with very high to very low quality, and first aid measures not performed but should have been performed

The overall quality of bystander first aid was evaluated as very high or high in 84/113 (74%) of the cases. The most frequently performed first aid measure was hypothermia prevention (64%). Overview of first aid measures is presented in Table 3 We found no significant difference in first aid quality between cases where video streaming was used or not (see Table 4). Figure 3 provides details on performance of each of the first aid measures with video streaming and with audio only.

**Table 3:** Overview of first aid measures and quality of performed first aid as assessed by on-scene ambulance personnel (*N*=113)

	**Airway management n, (%)**			**Bleeding control n, (%)**			**Recovery position n, (%)**			**Hypothermia prevention n, (%)**			**Overall quality of first aid measures n, (%)**		
	**Total**	**Audio only**	**Video streaming**	**Total**	**Audio only**	**Video streaming**	**Total**	**Audio only**	**Video streaming**	**Total**	**Audio only**	**Video streaming**	**Total**	**Audio only**	**Video streaming**
**Not needed**	105 (93)	93 (92)	12 (100)	81 (72)	75 (74)	6 (50)	85 (75)	78 (77)	7 (58)	24 (21)	21 (21)	3 (25)			
**Needed, not performed**	2 (2)	2 (2)		6 (5)	5 (5)	1 (8)	3 (3)	3 (3)		17 (15)	15 (15)	2 (17)			
**Needed, performed**	6 (6)	6 (6)		26 (23)	21 (21)	5 (41)	25 (22)	20 (20)	5 (42)	72 (64)	65 (65)	7 (58)			
**Quality when performed:**															
Very poor quality							1 (1)						1 (1)	1 (1)	
Poor quality				2 (2)	2 (2)			1 (1)					2 (2)	2 (2)	
Moderate quality	1 (1)	1 (1)		6 (5)	5 (5)	1 (8)	4 (4)	2 (2)	2 (17)	15 (13)	14 (14)	1 (8)	26 (23)	24 (24)	2 (17)
High quality	2 (2)	2 (2)		9 (8)	8 (8)	1 (8)	14 (13)	14 (14)		37 (33)	31 (31)	6 (50)	44 (39)	37 (37)	7 (58)
Very high quality	3 (3)	3 (3)		9 (8)	6 (6)	3 (25)	6 (5)	3 (3)	3 (25)	20 (18)	20 (20)		40 (35)	37 (37)	3 (25)

**Table 4:** Association between first aid measures performed by bystander with high quality (outcome variable) and video streaming, adjusted for by bystander background, recognized need first aid by dispatcher during the call, sex of patient and age of patient (*N*=113)

**Independent variables**	**First aid measures performed with high quality**	**Crude OR (95% CI)**	***P*-value**	**Adjusted OR(95% CI)**^a^	***P*-value**
**Calls where video streaming was used by dispatcher**					
** Yes**	10/12 (83%)	1.82(0.38 – 8.86)	0.46	4.30 (0.74 – 24.94)	0.10
** No (ref)**	74/101 (73%)	1		1	
**Bystander background**					
** Trained (police/fire brigade/civilian first aid responder)**	41/46 (89%)	4.5(1.60 – 13.13)	<0.05	8.0(2.33 – 27.49)	<0.05
** Lay person (ref)**	43/67 (64%)	1		1	
**Need for first aid recognized by dispatcher during the call**					
** Yes**	43/59 (73%)	0.85(0.37 – 1.99)	0.71	1.66(0.56 – 4.90)	0.36
** No (ref)**	41/54 (76%)	1		1	
**Sex of patient**					
** Male**	50/65 (77%)	1.41(0.59 – 3.21)	0.47	1.37(0.49 – 3.88)	0.55
** Female (ref)**	34/48 (71%)	1		1	
**Age of patient**					
** 0-18**	14/19 (77%)	1.65(0.46 – 5.96)	0.45	1.71(0.39 – 7.52)	0.48
** 19-30**	13/17 (76%)	1.91(0.49 – 7.49)	0.35	2.22(0.45 – 11.08)	0.33
** 31-50**	11/15 (73%)	1.62(0.41 – 6.47)	0.47	0.84(0.17 – 4.24)	0.83
** 51-70**	29/35 (83%)	2.84(0.88 – 9.22)	0.08	3.96(1.08 – 14.52)	<0.05
** 71 < (ref)**	17/27 (63%)	1		1	

^a^Adjusted for bystander background, need for first aid recognized by dispatcher during the call, sex of patient, age of patient

Trained bystanders performed first aid with higher quality. With crude logistic regression analysis, the bystanders who were trained were 4.5 times more likely to perform first aid of high quality compared to untrained bystanders (OR 4.5, 95% CI 1.60-13.13, *p*-value <0.05) (Table 4).

## Discussion

The dispatchers did not use video streaming frequently during medical emergency calls. We expected that the use of video streaming in calls regarding injured patients would be higher as dispatchers in previous studies have suggested video streaming useful for this patient group [8, 11]. We found that dispatchers were more likely to recognize the need for first aid, and to help the bystanders make adjustments to performed measures when video streaming was used. This suggests that video streaming may be a valuable tool for the dispatchers to increase the frequency of bystander first aid in trauma. However, with our sample, we could not demonstrate a significant association between video streaming and bystander first aid quality.

A low sensitivity for dispatchers recognizing need for first aid measures for injured patients has previously been seen [3]. In our study, the need for first aid was recognized by the dispatcher during the call in 52% of the total cases. Having audio only as a source of information during a medical emergency call can be challenging for the dispatcher [12]. Research on video streaming involving all reasons for contact has shown that it can change the dispatcher’s perception of the situation of the call [8, 11]. It is therefore plausible that video streaming can aid the dispatcher to more often recognize injured patients who has a need for first aid. Our results also imply that the dispatcher more often becomes aware of performed first aid measures which need adjustments when video streaming is used. However, there are several possible confounders to these results. The dispatcher might choose to use video streaming more often in the cases where they understand that the patient has a need for first aid. Our results do not contain information about whether video streaming was activated before or after first aid was addressed during the call.

We did not find that the use of video streaming by dispatcher was associated with increased quality of bystander first aid (OR=1.82, 95% CI 0.38-8.86). However, both the small number of cases in the group where video streaming was used, and the high overall quality of bystander first aid could have impacted the result. Studies which have looked at video streaming and bystander CPR-quality has shown both improvement in compression technique and increased survival to discharge [9, 13–15]. This suggests that video streaming should have a positive effect on bystander first aid in trauma, for example, assessment of severity of external bleeding and technique for airway management. Indeed, we did find that dispatchers were more likely to adjust the first aid measures performed on scene when they used video streaming. However, due to the study’s sample size, research with adequate power would be required to establish any association between bystander first aid quality and video streaming. Furthermore, bystander first aid overall holds a high quality, and trained bystanders performed first aid with the highest quality. These findings are also seen in a previous study by Bakke et al. [4]. It is possible that improvement of first aid quality with video streaming is challenging to demonstrate because bystander first aid already holds such a high quality, and that video streaming would have a higher impact in regions where the public is less trained in first aid.

### Strengths and limitations

Our data concerning first aid measures performed by bystanders is based on a subjective assessment by ambulance personnel. Bystander first aid is difficult to assess, and no validated tools exist to rate bystander first aid. For this reason, we developed the FAQA tool based on a previous study by Bakke et.al [4]. We have previously conducted a validation study of the FAQA tool, and demonstrated that the FAQA-tool is efficient for collecting information on bystander first aid, and reduces bias connected to the issue of subjectivity when rating first aid quality [10].

The sample size in our study did not meet our estimated sample size computed prior to inclusion. Our estimated sample size was based on the literature. Unfortunately, fewer patients than anticipated were included, and the dispatchers used video streaming more seldom for the study population than we expected. This study is a cross-sectional study and not a randomized controlled trial. For this reason, there might be a difference in background variables between the group where video streaming was used and the group where video streaming was not used. Because of the low number in the video streaming group, it was not possible to check for confounding variables that might have impacted on the study’s outcome variables.

The small number of patients included in our study could be due to several factors. We cannot exclude the possibility of missed inclusions and thus a risk of selection bias. Ideally, the study should have had regular controls to check for eligible patients who were not included. This was not possible, partly due to the nature of the ambulance and EMCC computer systems. Audio logs for 11 of the included patients were also missing. Nevertheless, we believe the patients included in our study are representative for patients with injuries in need for the first aid measures. Factors improving generalizability is recruitment of patients from three different study areas and a systematic training of ambulance personnel in the different areas.

Even though the group of cases where video streaming was used is very small, the number was sufficient to perform a univariate regression logistic analysis. The main problem with a small sample is a risk of type II error resulting in a missed recognition of an effect of the intervention as statistically significant. According to our study, the odds that the dispatcher recognizes a need for first aid were higher if video streaming was used during the call (OR=5.30, *p*-value <0.05). This association is statistically significant regardless of the small sample in the group where video streaming was used. The study population is too small to show significant associations for the hypothesis regarding effect of video streaming on bystander first aid quality, as indicated in the data. This secondary outcome would need a sufficient sample size to be retested for an eventual association. In addition, we were not able to adjust our statistical model for possible confounders.

This is the first study which has investigated the association between video streaming during medical emergency calls and first aid for injured patients. Research on the effects of video streaming during medical emergency calls is still sparse [16]. Even though the number of cases where video streaming was used by dispatcher is small and that the study has limitations, we believe that our main findings are important notions for further research on the topic.

## Conclusions

The need for first aid measures was recognized more often during the medical emergency call when the dispatcher used video streaming, and the use of video streaming may therefore increase frequency of bystander first aid. First aid performed with high quality by bystander did not have a significant association with the use of video streaming during calls. However, the study also shows that video streaming in medical emergency calls regarding injured patients is not frequently used by dispatchers in Norway.

Our hypothesis could be re-tested when video streaming in EMCCs is more prevalent, in order to ensure a better sample size.

### Supplementary Information

Supplementary Material 1.

Supplementary Material 2.

Supplementary Material 3.

Supplementary Material 4.

Supplementary Material 5.

## Data Availability

Data are available upon reasonable request.

## Abbreviations

EMCC: Emergency medical communication center
CPR: Cardiopulmonary resuscitation

## References

[1] Gerwing J, Steen-Hansen JE, Mjaaland T, Jensen BF, Eielsen O, Thomas OMT (2021). Evaluating a training intervention for improving alignment between emergency medical telephone operators and callers: a pilot study of communication behaviours. Scand J Trauma Resusc Emerg Med..

[2] Leonardsen AC, Ramsdal H, Olasveengen TM, Steen-Hansen JE, Westmark F, Hansen AE (2019). Exploring individual and work organizational peculiarities of working in emergency medical communication centers in Norway- a qualitative study. BMC Health Serv Res..

[3] Bakke HK, Steinvik T, Ruud H, Wisborg T (2017). Effect and accuracy of emergency dispatch telephone guidance to bystanders in trauma: post-hoc analysis of a prospective observational study. Scand J Trauma Resusc Emerg Med..

[4] Bakke HK, Steinvik T, Eidissen SI, Gilbert M, Wisborg T (2015). Bystander first aid in trauma - prevalence and quality: a prospective observational study. Acta Anaesthesiol Scand..

[5] Murad MK, Husum H (2010). Trained lay first responders reduce trauma mortality: a controlled study of rural trauma in Iraq. Prehosp Disaster Med..

[6] Oliver GJ, Walter DP, Redmond AD (2017). Prehospital deaths from trauma: Are injuries survivable and do bystanders help?. Injury..

[7] Tannvik TD, Bakke HK, Wisborg T (2012). A systematic literature review on first aid provided by laypeople to trauma victims. Acta Anaesthesiol Scand..

[8] Idland S, Iversen E, Brattebo G, Kramer-Johansen J, Hjortdahl M (2022). From hearing to seeing: medical dispatchers' experience with use of video streaming in medical emergency calls - a qualitative study. BMJ Open..

[9] Linderoth G, Rosenkrantz O, Lippert F, Ostergaard D, Ersboll AK, Meyhoff CS (2021). Live video from bystanders' smartphones to improve cardiopulmonary resuscitation. Resuscitation..

[10] Idland S, Kramer-Johansen J, Bakke HK, Hjortdahl M (2023). Assessing bystander first aid: development and validation of a First Aid Quality Assessment (FAQA) tool. BMC Emerg Med..

[11] Linderoth G, Lippert F, Ostergaard D, Ersboll AK, Meyhoff CS, Folke F (2021). Live video from bystanders' smartphones to medical dispatchers in real emergencies. BMC Emerg Med..

[12] Ek B, Svedlund M (2015). Registered nurses' experiences of their decision-making at an Emergency Medical Dispatch Centre. J Clin Nurs..

[13] Stipulante S, Delfosse AS, Donneau AF, Hartsein G, Haus S, D'Orio V (2016). Interactive videoconferencing versus audio telephone calls for dispatcher-assisted cardiopulmonary resuscitation using the ALERT algorithm: a randomized trial. Eur J Emerg Med..

[14] Ecker H, Lindacher F, Adams N, Hamacher S, Wingen S, Schier R (2020). Video-assisted cardiopulmonary resuscitation via smartphone improves quality of resuscitation: A randomised controlled simulation trial. Eur J Anaesthesiol..

[15] Lee SY, Song KJ, Shin SD, Hong KJ, Kim TH (2020). Comparison of the effects of audio-instructed and video-instructed dispatcher-assisted cardiopulmonary resuscitation on resuscitation outcomes after out-of-hospital cardiac arrest. Resuscitation..

[16] Sykora R, Peran D, Renza M, Bradna J, Smetana J, Duska F (2022). Video Emergency Calls in Medical Dispatching: A Scoping Review. Prehosp Disaster Med..

