# Supplementary material for: Can video streaming improve first aid for injured patients? A prospective observational study from Norway
Source: BMC Emerg Med. 2024 May 28;24:89. doi: 10.1186/s12873-024-01010-0 (PMC11131190; doi:10.1186/s12873-024-01010-0)
Supplement: Supplementary file 1 — Supplementary Material 1. [file 12873_2024_1010_MOESM1_ESM.docx]

Additional file 1

Characteristics of cases where video streaming was used by dispatcher.

|  | Problem | Bystander background | First aid measure (s) performed by bystander | Overall quality of first aid measures | Need for first aid recognized by dispatcher during the call |
| --- | --- | --- | --- | --- | --- |
| Case 1 | Injury/accident, acute | Lay person | Bleeding control | High quality | Yes |
| Case 2 | Injury/accident, acute | Lay person | Bleeding control, hypothermia prevention | High quality | Yes |
| Case 3 | Injury/accident, acute | Lay person | Bleeding control, hypothermia prevention | Moderate quality | Yes |
| Case 4 | Injury/accident, acute | Police or fire brigade | Hypothermia prevention | High quality | Yes |
| Case 5 | Injury/accident, acute | Lay person | Bleeding control | Very high quality | Yes |
| Case 6 | Injury/accident, acute | Lay person | Recovery position, hypothermia prevention | Very high quality | Yes |
| Case 7 | Injury/accident, urgent | Lay person | Hypothermia prevention | High quality | No |
| Case 8 | Injury/accident, urgent | Lay person | Bleeding control, hypothermia prevention | High quality | Yes |
| Case 9 | Unconscious person, acute | Lay person | Recovery position | Moderate quality | Yes |
| Case 10 | Unconscious person, acute | Lay person | Recovery position | Very high quality | Yes |
| Case 11 | Undetermined problem, acute | Lay person | Recovery position | High quality | Yes |
| Case 12 | Injury/accident, urgent | Lay person | Recovery position, hypothermia prevention | High quality | No |
