# Supplementary material for: Can video streaming improve first aid for injured patients? A prospective observational study from Norway
Source: BMC Emerg Med. 2024 May 28;24:89. doi: 10.1186/s12873-024-01010-0 (PMC11131190; doi:10.1186/s12873-024-01010-0)
Supplement: Supplementary file 3 — Supplementary Material 3. [file 12873_2024_1010_MOESM3_ESM.pdf]

Nettskjema

The First Aid Quality Assessment (FAQA) tool for evaluation of bystander first aid by ambulance personnel

Ambulance personnel fill out the questionnaire for all cases where:

the patient has an **injury**

there is more than one person present in addition to the patient (a potential first aid performer)

one or more of the following first aid measures have been conducted or should have been conducted before the arrival of the ambulance personnel:

-airway management
- external bleeding control
-recovery position
-hypothermia prevention

Incident number

The incident number of the mission

Sex of patient

Male

Female

Age of patient

Estimated age or exact age

Exact

Estimated

Who is the bystander(s) performing first aid?

Register whether the bystander performing first aid is from the police, fire brigade or a civilian first aid responder who has been called to the incident

Police or fire brigade

Civilian first aid responder (not police or fire brigade)

Lay persons

Airway management: Did bystander attempt airway management on the patient?

Examples: Chin lift, jaw thrust

Performed

Not performed, the patient was NOT in need of this measure

Not performed, but the patient WAS in need of this measure

Quality of airway management

Dette elementet vises kun dersom alternativet «Performed» er valgt i spørsmålet «Airway management: Did bystander attempt airway management on the patient?»

How would you evaluate the quality of bystander’s airway management of the patient?

Very poor

Poor

Moderate

High

Very high

External bleeding control: Did bystander attempt bleeding control on the patient?

Examples: Direct pressure, lifting of extremity, stop the bleed with e.g. cloth or with torniquet

Performed

Not performed, the patient was NOT in need of this measure

Not performed, but the patient WAS in need of this measure

Quality of bleeding control

Dette elementet vises kun dersom alternativet «Performed» er valgt i spørsmålet «External bleeding control: Did bystander attempt bleeding control on the patient?»

How would you evaluate the quality of bystander’s attempt on bleeding control?

Very poor

Poor

Moderate

High

Very high

Recovery position: Did bystander attempt to put the patient in recovery position?

Performed

Not performed, the patient was NOT in need of this measure

Not performed, but the patient WAS in need of this measure

Quality of recovery position

Dette elementet vises kun dersom alternativet «Performed» er valgt i spørsmålet «Recovery position: Did bystander attempt to put the patient in recovery position?»

How would you evaluate the quality of bystander’s attempt on putting the patient in recovery position?

Very poor

Poor

Moderate

High

Very high

Hypothermia prevention: Did bystander attempt to perform measures on the patient to prevent hypothermia?

Examples: isolating layers between the patient and the ground, shield the patient by bringing him/her inside e.g. a house or a car, cover the patient with e.g. wind canvas/sleeping bag/blanket, removal of patient’s moist/wet clothing.

Performed

Not performed, the patient was NOT in need of this measure

Not performed, but the patient WAS in need of this measure

Quality of measures for hypothermia prevention

Dette elementet vises kun dersom alternativet «Performed» er valgt i spørsmålet «Hypothermia prevention: Did bystander attempt to perform measures on the patient to prevent hypothermia?»

How would you evaluate the quality of bystander’s attempt to prevent the patient from hypothermia?

Very poor

Poor
Moderate
High
Very high

Overall quality of first aid measures

How would you evaluate the overall quality of bystander’s performance of lifesaving first aid measures on the patient?

Very poor
Poor
Moderate
High
Very high
