# Supplementary material for: Can video streaming improve first aid for injured patients? A prospective observational study from Norway
Source: BMC Emerg Med. 2024 May 28;24:89. doi: 10.1186/s12873-024-01010-0 (PMC11131190; doi:10.1186/s12873-024-01010-0)
Supplement: Supplementary file 4 — Supplementary Material 4. [file 12873_2024_1010_MOESM4_ESM.pdf]

Nettskjema

Audio log data collection

Mission number

Mission number for event

Was video streaming used by dispatcher during the call?

- Yes
- No
- Tried to use but did not work

Does the dispatcher recognize that the patient has a need for first aid?

Relevant first aid measures are airway management, external bleeding control, recovery position and hypothermia prevention

- Yes, and the caller has started to perform first aid
- Yes, and the dispatcher asks the caller to perform first aid
- No, need is not recognized, first aid measures are not performed (no measures are mentioned during the call),
- The caller tells the dispatcher that they have started to perform first aid before the dispatcher has asked
- Uncertain

Time from call start with the caller until the dispatcher recognizes that the patient has a need for first aid.

Dette elementet vises kun dersom alternativet «Yes, and the caller has started to perform first aid eller Yes, and the dispatcher asks the caller to perform first aid eller The caller tells the dispatcher that they have started to perform first aid before the dispatcher has asked» er valgt i spørsmålet «Does the dispatcher recognize that the patient has a need for first aid?»

Measured from the first word in the sentence where the dispatcher asks the caller to take action OR asks if action has been taken OR the caller themselves says they have started action. Written as minutes:seconds

Which first aid measures does the dispatcher recognize that the patient needs

Identified by the dispatcher asking if the action has been taken OR asking the caller to take the action OR the caller themselves stating that the action has been started. Evaluate for all four first aid measures

Airway management (e.g. jawlift)

- Yes
- No
- Not relevant/not necessary

External bleeding control

- Yes
- No
- Not relevant/not necessary

Recovery position

Yes

No

Not relevant/not necessary

Hypothermia prevention

Yes

No

Not relevant/not necessary

Are any first aid measures performed by the public corrected by the AMK dispatcher during the call?

The dispatcher asks the caller to correct one or more first aid measures. Evaluate for all four first aid measures

Airway management (e.g. jawlift)

Yes

No

Not applicable, this measure was never attempted on the patient.

External bleeding control

Yes

No

Not applicable, this measure was never attempted on the patient.

Recovery position

Yes

No

Not applicable, this measure was never attempted on the patient.

Hypothermia prevention

Yes

No

Not applicable, this measure was never attempted on the patient.

Was the need for correction of the first aid measure recognized because of video?

Dette elementet vises kun dersom alternativet «Yes eller Tried to use but did not work» er valgt i spørsmålet «Was video streaming used by dispatcher during the call?»

Yes

No

Uncertain

Free text field

Enter if you think there is other important information that emerges from the conversation
