# Supplementary material for: Can video streaming improve first aid for injured patients? A prospective observational study from Norway
Source: BMC Emerg Med. 2024 May 28;24:89. doi: 10.1186/s12873-024-01010-0 (PMC11131190; doi:10.1186/s12873-024-01010-0)
Supplement: Supplementary file 5 — Supplementary Material 5. [file 12873_2024_1010_MOESM5_ESM.pdf]

Nettskjema

Data fra EMCC patient chart

Mission number

Mission number for event

Index-criteria

Index criteria for event. Written as e.g. A.08.04

Social security number

Social security number for the patient

Date of the call

Written as dd.mm.yyyy

Time of day the call is answered

Written as hh:mm:ss

Geographic location for event

Closest specific place that can be retrieved from chart

Is video streaming recorded as used?

As a separate measure or described in the free text field

- Yes
- No

Resources on site

Which and how many resources were alerted about the incident?

Ambulance

- 0
- 1
- 2
- 3

Physician

- 0
- 1
- 2
- 3

Air ambulance

0
1
2
3

Ambulance boat

0
1
2
3

Single paramedic unit

0
1
2
3

ATV/snowmobile

0
1
2
3

Ski patrol

0
1
2
3

First aid responder

0
1
2
3

Delivery location for patient

Hospital
Out-of-hours emergency clinic
GP office
The patient was asked to use own transport/or no need for follow-up
The patient does not wish to go with the ambulance
Deceased

Time of activation of first resource

Written as hh:mm:ss

Nettskjema

Time the first resource arrived at the scene
Written as hh:mm:ss

Time the first resource departed
Written as hh:mm:ss

Time the patient is delivered at the destination
Written as hh:mm:ss. If the patient does not get transport from the emergency services, write 00:00:00

Free text fiels
Other relevant information from chart about first aid or video streaming
